# Biorthogonal-Wavelet-Based Method for Numerical Solution of Volterra Integral Equations

**DOI:** 10.3390/e21111098

**Published:** 2019-11-10

**Authors:** Mutaz Mohammad

**Affiliations:** Department of Mathematics and Statistics, College of Natural and Health Sciences, Zayed University, 144543 Abu Dhabi, UAE; Mutaz.Mohammad@zu.ac.ae; Tel.: +971-2-599-3496

**Keywords:** Volterra integral equations, multiresolution analysis, oblique extension principle, pseudo-splines, biorthogonal wavelets, quasi-affine systems

## Abstract

Framelets theory has been well studied in many applications in image processing, data recovery and computational analysis due to the key properties of framelets such as sparse representation and accuracy in coefficients recovery in the area of numerical and computational theory. This work is devoted to shedding some light on the benefits of using such framelets in the area of numerical computations of integral equations. We introduce a new numerical method for solving Volterra integral equations. It is based on pseudo-spline quasi-affine tight framelet systems generated via the oblique extension principles. The resulting system is converted into matrix equations via these generators. We present examples of the generated pseudo-splines quasi-affine tight framelet systems. Some numerical results to validate the proposed method are presented to illustrate the efficiency and accuracy of the method.

## 1. Introduction

Many natural science problems are modeled by Volterra integral equations, which therefore has brought them much attention from scientists in numerical analysis. Yet, many numerical schemes used wavelet representation to numerically solve some integral equations. However, some approximations work better with redundant expansions such as the biorthogonal wavelet (or simply, framelet) expansions. The redundancy property of framelets has been used for many applications in science and engineering disciplines, for example, in the analysis of the Gibbs phenomenon and numerical solutions of various types of integral equations (see, e.g., [1,2,3,4,5,6]), in time–frequency theory for image analysis, multifilter designs in electrical engineering, the theory of nonshift and shift-invariant spaces, and many other areas (see, e.g., [7,8,9,10,11,12,13]. It is known that the approximation accuracy improved via tight framelets due to their redundancy. Note that, in orthonormal expansion analysis, the redundancy is missing. Therefore, we have more freedom in building efficient and accurate recovery.

The aim of this paper is to present a numerical method by using a specific type of framelets generated using the unitary and oblique extension principles for approximating the solution of Volterra integral equations defined by

$$u\left(x\right)=f\left(x\right)+\lambda \int_{a}^{x}K(x,t)u(t)dt.$$

It is difficult, in most cases, to find the solution of the Volterra integral equations analytically. The collocation-type method is well known as an accurate numerical technique for integral equations.

We use a new and accurate method that generalizes the wavelet-collocation method used in the literature. We call it the framelet-collocation method.

Our paper is organized as follows. In Section 2, we provide some preliminary background on redundant systems (tight frames), their notations, and function expansion. Section 3 provides some principles in the construction of pseudo-spline quasi-affine tight framelet systems using the oblique extension principles. We then start the presentation of matrix assembly for solving Volterra integral equations based on the collocation-type pseudo-spline-quasi-affine-tight-framelets-based method in Section 4. We further test our method on a numerical example and some graphical illustrations in Section 5. In Section 6, we conclude with some comments.

## 2. Preliminary Results

The expansion of a function is not limited, in general, to a specific form, and we can have a redundancy for a given representation, for example, in the expansion generated via tight frames. The idea of the frame sequence was introduced in Ref. [14], where frames were used in the mathematical construction in the analysis of non-harmonic Fourier expansions. In Ref. [15], Daubechies presented these sets of tight frames of $L^{2}\left(R\right)$ in some applications of signal analysis.

**Definition** **1.** *A sequence ${\left\{f_{k}\right\}}_{k=1}^{\infty }$ of generators in the space $L^{2}\left(R\right)$ is called a framelet for $L^{2}\left(R\right)$ if *∃* numbers A,B>0 such that*

$${A∥f∥}^{2}\leq \sum_{k=1}^{\infty }{\left|\langle f,f_{k}\rangle \right|}^{2}\leq B{∥f∥}^{2},\;\forall f\in L^{2}\left(R\right).$$

The constants A,B are called framelet bounds. A framelet is called tight if it is possible to have A=B as a framelet bound. In fact, framelets are extensions of orthonormal bases. The space $L^{2}\left(R\right)$ is the set elements g(x) such that

$$\begin{array}{c} {∥g∥}_{L^{2}\left(R\right)}={\left(\int_{R}{\left|g\left(x\right)\right|}^{2}\right)}^{1/2}<\infty . \end{array}$$

Let $f\in L^{2}\left(R\right)$, then the dilation and translation functions, *D* and *T*, are defined by $Df\left(x\right)=\sqrt{2}f\left(2x\right)$ and $T_{a}f\left(x\right)=f\left(x-a\right)$ for a∈R, respectively. Note that for j∈Z, we have $T_{a}D^{j}=D^{j}T_{2^{j}a}$ and $D^{j}T_{a}=T_{2^{-j}a}D^{j}$. Define

$$\ell_{2}\left(Z\right)=\left\{h\left[k\right]:\sqrt{\sum_{k=-\infty }^{\infty }{\left|h\left[k\right]\right|}^{2}}<\infty ,k\in Z\right\}.$$

For $f\in L^{2}\left(R\right)$, the Fourier transform and its inverse are defined, respectively, by

$$\hat{f}\left(\xi \right)=\int_{R}f\left(t\right)\;e^{-i\xi t}dt,\;\xi \in R$$

and

$$f\left(x\right)=\frac{1}{2\pi }\int_{R}\hat{f}\left(\xi \right)\;e^{i\xi x}d\xi ,\;x\in R.$$

**Definition** **2.** *Let ϕ be a compactly supported function in $\in L^{2}\left(R\right)$. Then ϕ is a refinable function if there exists a sequence $h_{0}\left[k\right]\in \ell_{2}\left(Z\right)$ such that the following equation is satisfied:*

(1) $$\phi \left(x\right)=2\sum_{k\in Z}h_{0}\left[k\right]\phi \left(2x-k\right).$$

*Here, we call $h_{0}$ the low mask filter of the refinable function ϕ.*

Note that we can write Equation (1) in the frequency domain as

(2) $$\hat{\phi }=\left(\hat{h_{0}}\hat{\phi }\right)\left(\cdot /2\right),$$

for some 2π-periodic $\hat{h_{0}}.$ In addition, it known that if ϕ∈$L^{2}\left(R\right)$ such that $\hat{\phi }\left(0\right)=1$, then we have, ${\hat{h}}_{0}\left(0\right)=1$ and ${\hat{h}}_{0}\left(\pi \right)=0$, (see, for example, Refs. [7,16,17]). Hence, ${\hat{h}}_{0}\left(\xi \right)$ can be written as

(3) $${\hat{h}}_{0}\left(\xi \right)={\left(\frac{1+e^{-i\xi }}{2}\right)}^{n}\tau \left(\xi \right),$$

where n∈N refers to the highest multiplicity of the roots of ${\hat{h}}_{0}\left(\pi \right)$ such that τ(ξ) is a polynomial of trigonometric functions with τ(0)=1. One can easily show that Equation (2) can be rewritten as

$$\hat{\phi }\left(\xi \right)=\prod_{j=1}^{\infty }{\hat{h}}_{0}\left(2^{-j}\xi \right).$$

Hence, by Equation (3), we have

(4) $$\hat{\phi }\left(\xi \right)=\prod_{j=1}^{\infty }{\left(\frac{1+e^{-i(2^{-j}\xi )}}{2}\right)}^{n}\prod_{j=1}^{\infty }\tau \left(2^{-j}\xi \right)={\left(\frac{1-e^{-i\xi }}{i\xi }\right)}^{n}\prod_{j=1}^{\infty }\tau \left(2^{-j}\xi \right).$$

We say that a function ψ has a vanishing moment of order *m* if

$$\int_{-\infty }^{\infty }x^{p}\psi \left(x\right)dx=0;\;\;p=0,\cdots ,m-1.$$

It is known that multiresolution analysis (MRA) is a tool to generate wavelet bases. To formulate the matrix from and the numerical solution of a given Volterra integral equation, we will study and use pseudo-spline quasi-affine tight framelets and their constructions that are derived from an MRA, and in particular, the oblique extension principle (OEP) in Ref. [17]. The interested reader should consult [18,19,20] and other related references for more details.

Define $\Psi ={\left\{\psi_{\ell }\right\}}_{\ell =1}^{r}\subset L^{2}\left(R\right)$ as

(5) $$\psi_{\ell }=2\sum_{k\in Z}h_{\ell }\left[k\right]\phi \left(2\cdot -k\right).$$

The sequence ${\left\{h_{\ell }\left[k\right],\;k\in Z\right\}}_{\ell =1}^{r}$ is called the high mask filter of Ψ. Equation (5) can be expressed in terms of its Fourier representation and is given by

$${\hat{\psi }}_{\ell }=\left(\hat{h_{\ell }}\hat{\phi }\right)\left(\cdot /2\right),\;\;\ell =1,\cdots ,r,$$

where ${\hat{h}}_{\ell }\left(\cdot \right)={\hat{h}}_{\ell }\left(\cdot +2\pi \right),\forall \ell =1,\cdots ,r.$

**Theorem** **1.** *Assume that ϕ is a refinable function in $L^{2}\left(R\right)$ with compact support. Let $h_{0}$ be its finitely supported low mask filter. Let*

$${\left\{h_{\ell }\left[k\right],\;k\in Z\right\}}_{\ell =1}^{r}$$

*be sequences with finite support. Then,*

(6) $$X\left(\Psi \right)=\left\{\psi_{\ell ,j,k}:\;\ell =1,\cdots ,r;j,k\;\mathrm{are}\mathrm{integers}\right\}$$

*generates a tight framelet system for $L^{2}\left(R\right)$ if the following equations are satisfied such that −π≤ξ≤π, where*

(7) $$\sum_{\ell =0}^{r}{\left|{\hat{h}}_{\ell }\left(\xi \right)\right|}^{2}=1\;\;and\;\;\sum_{\ell =0}^{r}{\hat{h}}_{\ell }\left(\xi \right){\hat{h}}_{\ell }\left(\xi +\pi \right)=0.$$

**Proof.**  See Ref. [17]. □

By Theorem 1, it can be concluded that for any function $f\in L^{2}\left(R\right)$, we have the tight framelet representation given by

(8) $$f=\sum_{\ell =1}^{r}\sum_{j\in Z}\sum_{k\in Z}\langle f,\psi_{\ell ,j,k}\rangle \psi_{\ell ,j,k}.$$

The expansion in Equation (8) is known as the best possible expansion of the function *f*, where it can be truncated by $S_{n}$, such that

(9) $$S_{n}f=\sum_{\ell =1}^{r}\sum_{j<n}\sum_{k\in Z}\langle f,\psi_{\ell ,j,k}\rangle \psi_{\ell ,j,k}.$$

We will use Equation (9) to find the numerical solution of a given Volterra integral equation using quasi-affine tight framelets generated by pseudo-spline functions.

## 3. Pseudo-Spline Quasi-Affine Tight Framelets

*B*-spline tight framelets are one of the most important framelets in the framelet family. They are interesting due to their simple structure and properties, e.g., they have compact support and are given by explicit and quite simple formulas in the time and Fourier domain. The smoothness of the *B*-spline increases as we increase *n*. It has an important role in applied numerical mathematics, geometric analysis, and many other areas (see, e.g., Refs. [21,22]).

**Definition** **3.** *The B-spline $N_{m+1}$ is defined by*

$$N_{m+1}\left(x\right)=\int_{0}^{1}N_{m}\left(x-t\right)dt,$$

*where $N_{1}\left(x\right)$ is the indicator function on the interval [0,1).*

**Definition** **4.** *Let m∈N. Then we define the B-spline $B_{m}$ by the following equation:*

$$B_{m}\left(x\right):=N_{m}\left(x+\frac{m}{2}\right).$$

*Hence, we define $B_{m}$ by*

$$B_{m+1}:=B_{m}*B_{1},m\in N,$$

*where $B_{1}\left(x\right)=\chi_{{}_{\left[-1/2,1/2\right]}}\left(x\right)$.*

**Figure 1:**
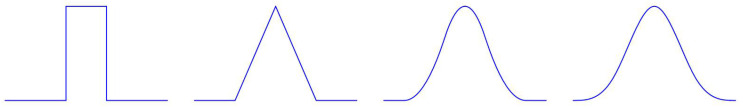
The *B*-splines $B_{m}$ for m=1,⋯,4, respectively.

For m=1,⋯,4, we plot the graphs of *B*-splines in Figure 1.

One can easily show that the Fourier transform of the *B*-spline, $B_{m}$, of order *m* is given by

$${\hat{B}}_{m}\left(\xi \right)={\left(\frac{\sin(\xi /2)}{\xi /2}\right)}^{m}\;\;and\;\;{\hat{h}}_{0}\left(\xi \right)=\cos^{m}\left(\xi /2\right).$$

Note that

$${\hat{N}}_{m}\left(\xi \right)={\hat{B}}_{m}\left(\xi -\frac{m}{2}\right)=e^{\frac{im\xi }{2}}{\hat{B}}_{m}\left(\xi \right),\;\mathrm{and}\;\mathrm{its}\;\mathrm{low}\;\mathrm{mask}\;\mathrm{filter}\;\mathrm{is}\;e^{\frac{im\xi }{2}}{\hat{h}}_{0}\left(\xi \right).$$

We refer the reader to [23] for more details.

The unitary extension principle (UEP) is known as a method to generate tight framelets via a refinable function. It is known that the MRA is a special case of the well known UEP. In addition, the UEP was extended to the OEP in [18,20] by finding a 2π-periodic function Θ. For any refinable function and to construct a tight framelet system, the non-negative function Θ, which is essentially bounded and continuous at zero such that Θ(0)=1, shall satisfy the following conditions:

$$\begin{array}{c} \Theta \left(2\xi \right)|{\hat{h}}_{0}{\left(\xi \right)|}^{2}=\Theta \left(\xi \right)-\sum_{\ell =1}^{r}{\left|{\hat{h}}_{\ell }\left(\xi \right)\right|}^{2};\\ \Theta \left(2\xi \right){\hat{h}}_{0}\left(\xi \right){\hat{h}}_{0}\left(\xi +\pi \right)+\sum_{\ell =1}^{r}{\hat{h}}_{\ell }\left(\xi \right){\hat{h}}_{\ell }\left(\xi +\pi \right)=0. \end{array}$$

**Definition** **5** ([24])**.** *Suppose that the conditions of the UEP hold for *Ψ*. Then, the quasi-affine system $X^{J}\left(\Psi \right)$ generated using *Ψ* is defined by*

$$X^{J}\left(\Psi \right)=\left\{\psi_{\ell ,j,k}:\ell =1,\cdots ,r;j,k\in Z\right\}$$

*such that*

$$\begin{array}{c} \psi_{\ell ,j,k}=\left\{\begin{array}{cc} 2^{j/2}\psi_{\ell }\left(2^{j}\cdot -k\right), & j\geq J\\ 2^{j}\psi_{\ell }\left(2^{j}\left(\cdot -k\right)\right), & j<J \end{array}\right.. \end{array}$$

Here, for our proposed method, we consider the system above for the case where J=0.

If ${\hat{h}}_{0}$ is the low mask filter of a given refinable function ϕ, then using the OEP, it is assumed [24] that

$$\Theta \left(\xi \right)-\Theta \left(2\xi \right)|{\hat{h}}_{0}{\left(\xi \right)|}^{2}\geq \Theta \left(2\xi \right){\left|{\hat{h}}_{0}\left(\xi +\pi \right)\right|}^{2}.$$

This condition helps to find the high mask filters of the required framelet system. Let ${\left|h\right|}^{2}=H$, where

$$H=\Theta \left(\xi \right)-\Theta \left(2\xi \right)|{\hat{h}}_{0}{\left(\xi \right)|}^{2}\geq \Theta \left(2\xi \right){\left|{\hat{h}}_{0}\left(\xi +\pi \right)\right|}^{2}$$

and

$${\left|\theta \right|}^{2}=\Theta .$$

Here, the square root is obtained by the spectral factorization in Ref. [7]. Assume that $c_{2},c_{3}$ are two 2π-periodic trigonometric functions/polynomials such that

$$|c_{2}{\left(\xi \right)|}^{2}+{\left|c_{3}\left(\xi \right)\right|}^{2}=1,\;\;c_{2}\left(\xi \right)\bar{c_{2}\left(\xi +\pi \right)}+c_{3}\left(\xi \right)\bar{c_{3}\left(\xi +\pi \right)}=0.$$

Then, we can find three high mask filters, namely,

$${\hat{h}}_{1}\left(\xi \right)=e^{i\xi }\theta \left(2\xi \right)\;\bar{{\hat{h}}_{0}\left(\xi +\pi \right)},\;\;{\hat{h}}_{2}\left(\xi \right)=c_{2}\left(\xi \right)h\left(\xi \right),\;\;4{\hat{h}}_{3}\left(\xi \right)=c_{3}\left(\xi \right)h\left(\xi \right),$$

with a standard choice of $c_{2}\left(\xi \right)=\left(1/\sqrt{2}\right)$ and $c_{3}\left(\xi \right)=\left(1/\sqrt{2}\right)e^{i\xi }$. If we consider the UEP rather than the OEP in the construction above, i.e., Θ=1, then we will use the assumption that

$$|{\hat{h}}_{0}{\left(\xi \right)|}^{2}\leq 1-{\left|{\hat{h}}_{0}\left(\xi +\pi \right)\right|}^{2}.$$

Define the high mask filters by

$${\hat{h}}_{1}\left(\xi \right)=e^{i\xi }\bar{{\hat{h}}_{0}\left(\pi +\xi \right)},\;\;{\hat{h}}_{2}\left(\xi \right)=\left(\sqrt{2}/2\right)h\left(\xi \right),\;\;{\hat{h}}_{3}\left(\xi \right)=e^{i\xi }{\hat{h}}_{2}\left(\xi \right).$$

Note that we can reduce the number of framelets from three to two with the new fundamental function 1−H, where

$${\hat{h}}_{1}\left(\xi \right)=e^{i\xi }\theta \left(2\xi \right)\bar{{\hat{h}}_{0}\left(\pi +\xi \right)},\;\;{\hat{h}}_{2}\left(\xi \right)=h_{0}\left(\xi \right)h\left(2\xi \right).$$

However, this will usually affect the framelet system by having less symmetry of the framelets or longer filters.

Let ϕ be as Equation (1), which generates an MRA ($V_{j}{)}_{j}$, and $W_{2}^{m}\left(R\right)$ be the Sobolev space. Then, X(Ψ) provides approximation order *m* if

$${∥f-S_{n}f∥}_{2}=O\left(2^{-nm}\right),\;\;\forall f\in W_{2}^{m}\left(R\right).$$

The approximation order of the truncated function $S_{n}$ was studied in [18,25]. It is known that the approximation orders rely on the behavior near zero of the function $\Lambda_{\phi }$, where

$$\Lambda_{\phi }=\sqrt{1-\frac{|\hat{\phi }{|}^{2}}{\left[\;\hat{\phi },\hat{\phi }\;\right]}}\;$$

and

$$\left[f,g\right]\left(\xi \right)=\sum_{k\in 2\pi Z}f\left(\xi +k\right)\bar{g(\xi +k)}.$$

Note that the refinable function ϕ satisfies *m*-Strang–Fix condition if the following equation is satisfied, where

$$\hat{\phi }\left(0\right)\neq 0,\;\;{\hat{\phi }}^{\left(j\right)}\left(2\pi k\right)=0,\;\;\;j\in 0,\cdots ,m-1,\;k\;\mathrm{is}\;a\;\mathrm{non}-\mathrm{zero}\;\mathrm{integer}.$$

It was proved in Ref. [25] that if the function ϕ provides approximation order *m*, then $\Lambda_{\phi }$ has a zero of order *m* at the origin. This means that $\Lambda_{\phi }$ has a zero of order *m* at ξ=0, and then $\hat{\phi }$ has a zero of order *m*. In addition, Jetter et al. in [26] showed that depending on the OEP construction, the truncated $S_{n}$ provides approximation order *m* iff $\left[\hat{\phi },\hat{\phi }\right]-{\left|\hat{\phi }\right|}^{2}=O\left(2^{2m}\right)$.

Daubechies, in Ref. [18], has proved that if the system X(Ψ) has a vanishing moment of order $m_{1}$ and ϕ has an approximation order *m*, then the approximation order of X(Ψ) is equal to the minimum of *m* and $2m_{1}.$ To have high approximation orders, we have to construct refinable functions where the Fourier transforms are very smooth at the origin. This leads to the well-known refinable functions, pseudo-splines, and their tight framelet generators.

Pseudo-splines provide us with a nice class of refinable and compactly supported functions. The first type was introduced in [18,27] to construct a special type of tight framelets and type II were introduced in [28] to construct tight framelets with specific properties of symmetry. In the frequency domain and for non-negative integers l,m such that l<m, pseudo-splines of type I (or **PS-I-(m,l)** and type II with order *m* and type *l* (or **PS-II-(m,l)**) can be defined by

$${}_{k}\hat{\phi }\left(\xi \right)=\prod_{i=1}^{\infty }{}_{k}{\hat{h}}_{0}\left(2^{-i}\xi \right)\;\mathrm{with}{\;}_{k}\hat{\phi }\left(0\right)=1,\;\mathrm{for}k=1,2,$$

where the low mask filter of the pseudo-splines of type I with order (m,l) is defined by

|1h^0(ξ)|2=cos2m(ξ/2)∑k=0lm+lksin2k(ξ/2)cos2(l−k)(ξ/2)

and the low mask filter of the pseudo-splines of type II with order (m,l) is defined by

2h^0(ξ)=cos2m(ξ/2)∑k=0lm+lksin2k(ξ/2)cos2(l−k)(ξ/2).

Note that if r=0, pseudo-splines of both types are *B*-splines. It is known that the smoothness of the pseudo-spline increases with *m* and decreases with *l* (see Ref. [28]). According to spectral factorization, or by using the Fejér–Riesz lemma (see [7]), the low mask filter of the pseudo-spline of type I is obtained by taking the square root of type II, i.e., ${}_{2}{\hat{h}}_{0}\left(\xi \right)={{|}_{1}{\hat{h}}_{0}\left(\xi \right)|}^{2}.$ In general, we have the following lemma.

**Lemma** **1.** *Assume that L(ξ) is a positive valued trigonometric polynomial given by*

$$f\left(\xi \right)=\sum_{m=0}^{M}a_{m}\cos\left(m\xi \right),\;\;\;with\;\;a_{m}\in R.$$

*Then *∃* a trigonometric polynomial g of order M, where*

$$g\left(\xi \right)=\sum_{m=0}^{M}b_{m}\;e^{im\xi },\;\;\;with\;\;b_{m}\in R,$$

*such that ${\left|g\left(\xi \right)\right|}^{2}=f\left(\xi \right)$.*

**Proof.**  See Ref. [7]. □

In Mallat’s construction (see Ref. [29]), it is shown that ${\hat{h}}_{0}\left(\xi \right)\bar{{\hat{h}}_{0}\left(\xi +\pi \right)}+{\hat{h}}_{1}\left(\xi \right)\bar{{\hat{h}}_{1}\left(\xi +\pi \right)}=0$, where ${\hat{h}}_{1}\left(\xi \right)=e^{i\xi }\;\bar{{\hat{h}}_{0}\left(\xi +\pi \right)}$, and that

(10) H(ξ)=cos2m(ξ)∑k=ℓ+1m−1m+ℓkcos2(ℓ−k)(ξ/2)sin2k(ξ/2).

If we take m=4 and ℓ=1, then we will get short filters compared with the general case. This is because of the form of H(ξ), where

H(ξ)=∑k=235kcos10−2k(ξ/2)sin2k(ξ/2)=10cos4(ξ/2)sin4(ξ/2).

In fact, we have the following fact.

**Proposition** **1.** *For non-negative integers l,m such that l<m, If ℓ=m−3, then*

H(ξ)=2m−3m−1cos2m−4(ξ/2)sin2m−4(ξ/2).

**Proof.**  As 2m−3m−2=2m−3m−1, then we have

H(ξ)=∑k=m−2m−12m−3kcos4m−6−2k(ξ/2)sin2k(ξ/2).

Therefore,

H(ξ)=2m−3m−2cos2m−4(ξ/2)sin2m−2(ξ/2)+2m−3m−1cos2m−2(ξ/2)sin2m−4(ξ/2)=2m−3m−1cos2m−4(ξ/2)sin2m−4(ξ/2).

 □

Using pseudo-splines of both types, we give some examples of quasi-affine tight framelets constructed via the OEP.

**Example** **1** **(PS-I** **-(4,1)).** *For the order (4,1), consider the pseudo-spline of type I, ${}_{1}\hat{\phi }\left(\xi \right)$. Then, its low mask filter can be given by ${|}_{1}{\hat{h}}_{0}{\left(\xi \right)|}^{2}=\cos^{8}\left(\xi /2\right)\left(1+4\sin^{2}\left(\xi /2\right)\right)$. Note that by using Lemma 1, we have*

$${}_{1}{\hat{h}}_{0}\left(\xi \right)=\frac{-e^{-i5\xi }{\left(1+e^{i\xi }\right)}^{4}\;\left(2+\left(-3+\sqrt{5}\right)\;e^{i\xi }\right)}{16(1-\sqrt{5})}.$$

*Define*

$${}_{1}{\hat{h}}_{1}\left(\xi \right)=e^{i\xi }{\;}_{1}{\hat{h}}_{0}\left(\xi +\pi \right),{\;}_{1}{\hat{h}}_{2}\left(\xi \right)=\frac{\sqrt{5}}{2}\sin^{2}\left(\xi \right),\;\mathrm{and}{\;}_{1}{\hat{h}}_{3}\left(\xi \right)=e^{i\xi }{\;}_{1}{\hat{h}}_{2}\left(\xi \right).$$

*Let $\Psi =\left\{{}_{1}\psi_{\ell };\;\ell =1,2,3\right\}$, where ${}_{1}{\hat{\psi }}_{\ell }={\;}_{1}{\hat{h}}_{\ell }{\left(\xi /2\right)}_{1}\hat{\phi }\left(\xi /2\right);\;\ell =1,2,3.$ Then the system $X^{0}\left(\Psi \right)$ forms a quasi-affine tight framelet for $L^{2}\left(R\right)$.*

**Figure 2:**
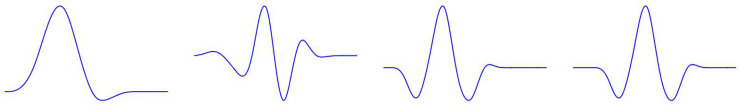
The type I pseudo-spline of order (4,1) along with its quasi-affine tight framelets, respectively.

The pseudo-spline of type I, with its corresponding quasi-affine tight framelets generated by using ${}_{1}\hat{\phi }\left(\xi \right)$ of order (4,1), is depicted in Figure 2.

**Example** **2** **(PS-II** **-(3,1)).** *For the order (3,1), consider the pseudo-spline of type II, ${}_{2}\hat{\phi }\left(\xi \right)$. Its low mask filter is given by*

$${}_{2}{\hat{h}}_{0}\left(\xi \right)=\cos^{6}\left(\xi /2\right)\left(1+3\sin^{2}\left(\xi /2\right)\right).$$

*Define*

$${}_{2}{\hat{h}}_{1}\left(\xi \right)=e^{-i\xi }{\;}_{2}{\hat{h}}_{0}\left(\xi +\pi \right),{\;}_{2}{\hat{h}}_{2}\left(\xi \right)=h\left(\xi \right)+e^{-i\xi }h\left(-\xi \right)$$

*and*

$${}_{2}{\hat{h}}_{3}\left(\xi \right)=e^{-i\xi }h\left(-\xi \right)-h\left(\xi \right),$$

*where*

$$\begin{array}{cc} H(\xi ) & =0.30108642578125305-0.2014160156250013e^{-2i\xi }-0.20141601562500128e^{2i\xi }+\\ \; & 0.05090332031249944e^{-4i\xi }+0.050903320312499445e^{4i\xi }+0.00024414062500026867e^{-6i\xi }+\\ \; & 0.00024414062500026867e^{6i\xi }-0.00027465820312493067e^{-8i\xi }-0.00027465820312493067e^{8i\xi }\\ \mathrm{and}\\ h(\xi ) & =-0.22813823298962+0.00139868605052e^{-2i\xi }+0.44712319189971e^{2i\xi }\\ \; & +0.00123930398199e^{-4i\xi }-0.2216229489426e^{4i\xi }. \end{array}$$

**Figure 3:**
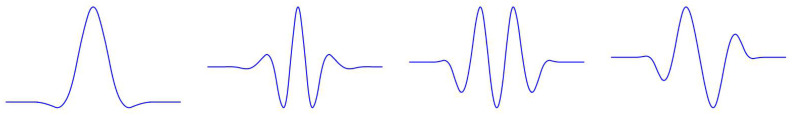
The pseudo-spline scaling function of type II with order (3,1) along with its quasi-affine tight framelets, respectively.

The pseudo-spline of type II with its corresponding quasi-affine tight framelets generated by ${}_{2}\hat{\phi }\left(\xi \right)$ of order (3,1) is depicted in Figure 3.

## 4. Matrix Assembly via Pseudo-Spline Quasi-Affine Tight Framelets

Consider the Volterra integral equation defined by

(11) $$u\left(x\right)=f\left(x\right)+\lambda \int_{a}^{x}K(x,t)u(t)dt,$$

where λ∈R, *f*, and *K* are given and known functions and *u* is an unknown function to be approximated. *K* is called the kernel of Equation (11). A function u(x) defined over [a,b] can be expressed by framelets as Equation (8). To find an approximate solution $u_{n}$ of Equation (11), we will truncate the framelet representation of *u* as Equation (9). Then,

(12) $$u\left(x\right)\approx u_{n}\left(x\right)=\sum_{\ell =1}^{r}\sum_{j<n}\sum_{k\in Z}c_{j,k}^{\ell }\psi_{\ell ,j,k},$$

where $c_{j,k}^{\ell }=\langle u_{n},\psi_{\ell ,j,k}\rangle$. Substituting Equation (12) into Equation (11) and by using the suitable collocation points to the truncated expansion, we have

(13) $$\sum_{\ell =1}^{r}\sum_{j<n}\sum_{i,k\in Z}c_{j,k}^{\ell }\psi_{\ell ,j,k}\left(x_{i}\right)=f\left(x\right)+\lambda \sum_{\ell =1}^{r}\sum_{j<n}\sum_{i,k\in Z}\int_{a}^{x}K\left(x_{i},t\right)c_{j,k}^{\ell }\psi_{\ell ,j,k}\left(t\right)dt.$$

Equation (13) can be simplified to a system of equations with the unknown coefficients $c_{j,k}^{\ell }$ given by

(14) $$\sum_{\ell =1}^{r}\sum_{j<n}\sum_{k\in Z}c_{j,k}^{\ell }M_{\ell ,j,k}\left(x_{i}\right)=f_{\ell ,j,k}\left(x_{i}\right),$$

where

(15) $$M_{\ell ,j,k}\left(x_{i}\right)=\psi_{\ell ,j,k}\left(x_{i}\right)-\lambda \int_{a}^{b}K\left(x_{i},t\right)\psi_{\ell ,j,k}\left(t\right)dt.$$

Now the unknown coefficients are determined by solving the resulting system of equations obtained from Equation (14), and then we get the approximate solution $u_{n}$. The absolute error for this formulation is defined by

$$e_{n}={∥u_{n}\left(x\right)-u\left(x\right)∥}_{2},\;\;x\in \left[a,b\right].$$

## 5. Numerical Performance

To validate the accuracy of our method, in this section we present the following example of Volterra integral equations. The numerical results obtained here using Mathematica software.

**Example** **3.** *We consider the following Volterra–Fredholm integral equation:*

$$u\left(x\right)=f\left(x\right)+\int_{0}^{x}e^{t}\cos\left(x\right)u\left(t\right)dt,$$

*where*

$$f\left(x\right)=e^{x}-\frac{1}{2}\cos\left(x\right)\left(e^{2x}-1\right)$$

*and the exact solution is $u\left(x\right)=e^{x}$.*

**Figure 4:**
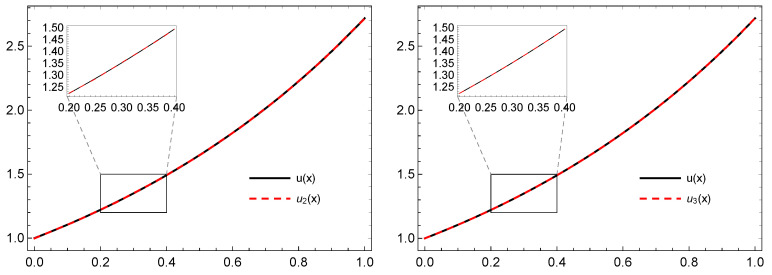
The graphs of *u* and $u_{n}$ for n=2,3, respectively, of Example 3 based on the **PS-I** **-(4,1)** framelet system.

**Figure 5:**
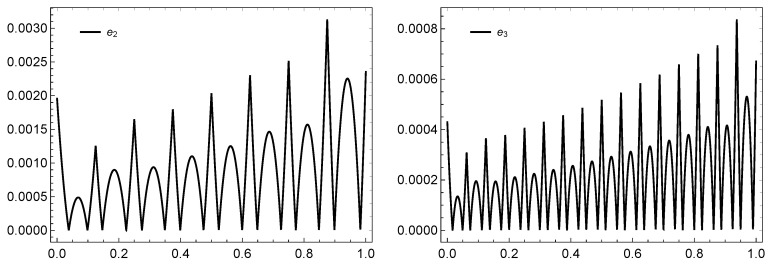
The graphs of the error for n=2,3, respectively, of Example 3 based on the **PS-I** **-(4,1)** framelet system.

In Table 1 and Table 2, the error $e_{n}$ for different values of *n* and the numerical values of the exact and approximated solution $u_{n}\left(x\right)$ when n=2 are computed, respectively. Some illustrations for the graphs of the exact and approximate solutions and the error are depicted in Figure 4 and Figure 5.

**Figure 6:**
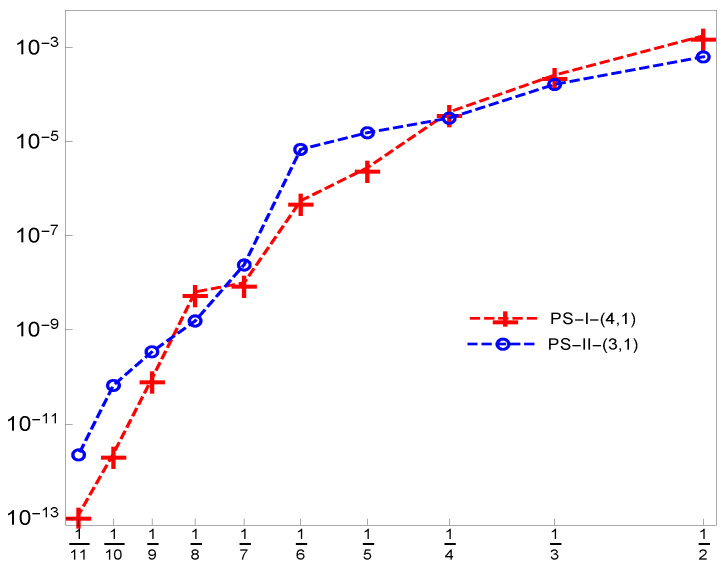
The rate of convergence of the proposed method for Example 3.

To validate the proposed method, we provide Figure 6 to show the rate of convergence of Example 3 in the log–log scale plot by using both systems of pseudo-spline quasi-affine tight framelets **PS-I** **-(4,1)** and **PS-II** **-(3,1)** generated using the OEP.

## 6. Conclusions

A collocation-type pseudo-spline-quasi-affine-framelet-based method is developed to numerically solve a given Volterra integral equation. This is an important research direction in the filed of framelet-based numerical schemes for integral equations. With a few orders of truncated partial sums, the results show that the proposed method is effective and accurate. It turns out that increasing the order of the truncated partial sums of the framelet system and its vanishing moments will dramatically increase the approximation orders as well as the accuracy of the solution. In addition, the accuracy orders of the approximated solution using both pseudo-spline quasi-affine tight framelet systems were close to each other, with slight differences and preferences to those with a higher order of *m*.

## Figures and Tables

**Table 1:** The error $e_{n}$ of Example 3 using different types of pseudo-spline framelet systems.

*n*	PS-I -(4,1)	PS-II -(3,1)
2	$1.08\times 10^{-3}$	$6.438\times 10^{-4}$
3	$2.63\times 10^{-4}$	$1.67\times 10^{-4}$
4	$4.32\times 10^{-5}$	3.21$\times 10^{-5}$
5	$2.83\times 10^{-6}$	0.57$\times 10^{-5}$
6	$5.58\times 10^{-7}$	6.93$\times 10^{-6}$
7	$1.01\times 10^{-8}$	2.44$\times 10^{-8}$
8	$6.44\times 10^{-9}$	1.56$\times 10^{-9}$
9	$9.35\times 10^{-11}$	$3.45\times 10^{-10}$
10	$2.35\times 10^{-12}$	$6.69\times 10^{-11}$

**Table 2:** Numerical results of the function $u_{n}$ of Example 3 using different types of pseudo-spline framelet systems and for a level n=2.

*n*	Exact	PS-I -(4,1)	PS-II -(3,1)
0.1	1.10517	1.105117381	1.105117134
0.2	1.22140	1.222175185	1.222124175
0.3	1.34986	1.350694516	1.340694516
0.4	1.49182	1.491882666	1.491802512
0.5	1.64872	1.646677909	1.646672532
0.6	1.82212	1.822082188	1.822194893
0.7	2.01375	2.015056716	2.015321421
0.8	2.22554	2.226976714	2.226776701
0.9	2.45960	2.459772459	2.459610020
1.0	2.71828	2.715929278	2.715989072
